# A Challenging Diagnosis of Ascites: A Case Report of Peritoneal Tuberculosis

**DOI:** 10.1155/2018/8136476

**Published:** 2018-10-23

**Authors:** Jose Ruiz, Maedeh Ganji, Catarina Canha, Carmen Isache

**Affiliations:** University of Florida-COM, Division of Internal Medicine, Jacksonville, FL, USA

## Abstract

Approximately 2 billion people, which is about one third of the world's population, are infected with tuberculosis (TB). Around 10% of infected people will develop active TB at one point in their lifetime. We present a rare case of a 68-year-old male who presented to the emergency department with a 2-week progressive dyspnea. In addition, the patient complained of generalized weakness, subjective fevers, and abdominal pain in the right upper quadrant. Ascites was noted on physical exam, and the patient underwent a diagnostic paracentesis with initial workup that was unrevealing for underlying etiology. Abdominal computed tomography was done, which revealed peritoneal carcinomatosis. He underwent omental biopsy which showed necrotizing granulomatous inflammation with rare acid-fast bacilli. Repeat biopsy was culture positive for mycobacterium tuberculosis complex. The patient was started on rifampin, isoniazid, pyrazinamide, and ethambutol with improvement of his symptoms days after treatment was started. This case demonstrates how the diagnosis of peritoneal tuberculosis can be elusive. Physicians must be aware of this disease and its behavior in high risk patients, also of the current diagnostic limitations.

## 1. Introduction

One third of the world's population, approximately 2 billion people, is infected with tuberculosis (TB). It is estimated that around 10% of infected people will develop active TB at one point in their lifetime. In the United States of America, it is estimated that around 18% of all tuberculosis cases are extrapulmonary, but peritoneal tuberculosis remains rare, accounting for only about 4.7% of all patients infected with TB. Immunocompromised patients are high risk for abdominal tuberculosis. High risk population includes patients with acquired immune deficiency syndrome, immigrants from areas where TB is endemic, Native Americans on reservations, the urban poor, and also the elderly. Some literature suggests also that alcoholic liver disease is a risk factor for peritoneal TB, but causality has not been established. We present a case of a 68-year-old male that presented with peritoneal tuberculosis in whom extensive workup was needed to achieve the correct diagnosis.

## 2. Case

A 68-year-old male with past medical history significant of end-stage renal disease on hemodialysis, hypertension, hyperlipidemia, diabetes mellitus type 2, cirrhosis, hepatitis C, chronic obstructive pulmonary disease, and benign prostate hyperplasia presented to the emergency department with a 2-week progressive dyspnea. In addition, the patient complained of generalized weakness, subjective fevers, and abdominal pain in the right upper quadrant. The initial blood tests showed a normal complete blood count, no renal function abnormalities, and no electrolyte abnormalities and aspartate transaminase (AST) of 19 IU/L, alanine transaminase (ALT) of 5 UI/L, albumin of 2.9 g/dL, total bilirubin of 1.4 mg/dL, and prothrombin time of 15.6 seconds. Hepatitis B surface antigen and human immunodeficiency virus (HIV) 1 and 2 antibodies were negative. CXR was obtained and revealed a large left pleural effusion. Diagnostic thoracentesis revealed an exudative pleural fluid per Light's criteria and negative cytology. Ascites was noted on physical exam, and the patient underwent a diagnostic paracentesis that revealed a serum ascites albumin gradient of <1.1, polymorphonuclear cell count <250/mm^3^, and negative culture. Repeat paracentesis was performed to rule out malignancy or tuberculosis as the cause of ascites. No malignant cells were found, adenosine deaminase (ADA) activity was 3.4 IU/L, and Ziehl–Neelsen stain, Lowenstein–Jensen cultures, and polymerase chain reaction (PCR) amplification were negative for mycobacteria. Decision for abdominal computed tomography was made, which revealed peritoneal carcinomatosis with multiple subcentimeter lesions in the liver, spleen, and adrenal glands. Interventional radiology was consulted, and an omental biopsy was obtained which showed necrotizing granulomatous inflammation with rare acid-fast bacilli (AFB). Repeat biopsy was done for tissue culture, and this was positive for *Mycobacterium tuberculosis* complex. He was started on treatment with rifampin, isoniazid, pyrazinamide, and ethambutol immediately after biopsy, based on very low resistance rates of *M. tuberculosis* complex in our area. This patient's mycobacterial isolate was sent to the local Department of Health laboratory, to confirm antimicrobial susceptibility. The patient's AFB sputum cultures have remained negative. The patient's systemic symptoms improved days after treatment was started.

## 3. Discussion

Peritoneal tuberculosis usually occurs due to reactivation of latent foci of infection in the peritoneum after hematogenous dissemination from a previous pulmonary infection. Interestingly, our patient had no prior known history of TB. Patients usually present with complaints of abdominal pain but can also have fevers, weight loss, fatigue, and generalized malaise. One will notice on physical exam that this particular patient presented with abdominal tenderness and ascites. The absence of chronic liver disease stigmata should increase suspicion for TB peritonitis. However, for patients with cirrhosis, such as our patient, due to more common causes of ascites, clinical suspicion is of extreme importance for the diagnosis.

The regional rate of TB reported in 2016 in the county of residence of this patient was 5.6 cases per 100,000 persons. 23% of the total number of reported TB cases in the region had extrapulmonary involvement. Our patient was not an immigrant. He was born and raised in the US and had no recent international travel history. His risk factors for TB infection included homelessness, history of intravenous drug use, diabetes mellitus, and end-stage kidney disease. Our patient did not have any family members with history of TB. He had been unemployed for past several years and denied any known TB contacts. No other recently reported TB cases were linked to him, to our knowledge. However, considering that he was homeless and had resided in several shelters, he had a high likelihood of being exposed to TB in the past.

Depending on the patient's past medical history one can put in place an idea of what to expect on results of the paracentesis. For example, in people with history of cirrhosis with ascites, spontaneous bacterial peritonitis should be high on the differential, but anchoring to that can lead to missing the right diagnosis. A positive tuberculin skin test or serum quantiferon may provide help in putting TB on the list of differential diagnosis, but is unable to differentiate between active and latent TB. Peritoneal fluid analysis, in cases of TB peritonitis, typically shows an elevated lymphocyte count with lymphocyte predominance, serum-ascitic albumin gradient <1.1, and high protein levels (>2.5 mg/dL) 5.

For abdominal tuberculosis, adenosine deaminase (ADA) has been shown to be useful in making the diagnosis, specifically when levels are above ≥30 U/L. In our case, ADA activity was extremely low. This is a finding that can be seen in cirrhotic patients, leading to increased difficulty in making the correct diagnosis. Cirrhotic patients have poor reticuloendothelial system function, resulting in lower than normal ADA levels. Other tests which can be useful in diagnosing peritoneal TB include culture of the fluid, Ziehl–Neelsen staining, and PCR assay for mycobacteria. In our case, all three were negative which can be explained by the low sensitivity of these tests, especially if mycobacterial burden in the ascitic fluid is low. Imaging is a useful tool to evaluate for ascites and peritoneal thickening. Abdominal sonography is used to guide needle aspiration of fluid and tissue biopsies. Computed tomography is more sensitive in detecting bowel thickening and abdominal lymphadenopathy.

The treatment of TB peritonitis is generally medical. It consists of the same six-month regimen used for pulmonary tuberculosis, initially with four first line agents, if regional resistance is low, then tailored down to only two agents based on the actual susceptibilities. *M. tuberculosis* complex was isolated from our patient's omental tissue culture. He was started initially on treatment with rifampin, isoniazid, pyrazinamide, and ethambutol, immediately after biopsy, based on very low resistance rates of *M. tuberculosis* complex in our area (regional incidence of multidrug resistant TB is only 1%, based on local Department of Health data). This patient's mycobacterial isolate was then sent to the local Department of Health laboratory, to confirm antimicrobial susceptibility; however, the result was not yet available at the time of submission of this case. His AFB sputum cultures were finalized as negative.

Surgical intervention is reserved for complications, such as bowel perforation, intestinal obstruction, fistulas, abscess, and hemorrhage. Early diagnosis and management can prevent unnecessary surgical intervention.

## 4. Conclusion

The case presented demonstrates how the diagnosis of peritoneal tuberculosis can be elusive. Physicians must be aware of the disease and its behavior in high risk patients and of the current diagnostic limitations. A combination of radiologic, microbiologic, and histopathological examinations helps achieve diagnostic accuracy and prevents a delay in treatment, which is associated with increased mortality and morbidity in patients with this disease.
